# Supplementary material for: Ecuadorian Provinces with High Morbidity and Mortality Rates Due to Asthma among the Working-Age Population: An Ecological Study to Promote Respiratory Health
Source: Int J Environ Res Public Health. 2024 Jul 11;21(7):909. doi: 10.3390/ijerph21070909 (PMC11276727; doi:10.3390/ijerph21070909)
Supplement: Supplementary file 1 [file ijerph-21-00909-s001.zip › ijerph-3041865-supplementary.pdf]

Supplementary Table 1: Occupational asthma cases in the Republic of Ecuador, 2016–2019

	**n**	**%**
**Total**	64	100
**Year**		
2016	5	7.8
2017	19	29.7
2018	17	26.6
2019	23	35.9
**Provinces**		
Azuay	3	4.7
El Oro	2	3.1
Esmeraldas	1	1.6
**Guayas**	**18**	**28.1**
Imbabura	1	1.6
Loja	3	4.7
Los Ríos	1	1.6
Manabí	2	3.1
Morona Santiago	1	1.6
Napo	1	1.6
Orellana	1	1.6
Pastaza	1	1.6
**Pichincha**	**23**	**35.9**
Santa Elena	1	1.6
Santo Domingo T.	4	6.3
Sucumbíos	1	1.6
**Sex**		
Men	37	57.8
Women	27	42.2
**Age**		
Average	45.9	
Standard deviation	10.2	
Minimum	30	
Maximum	67	

Source: General Labour Risk Insurance, Ecuadorian Institute of Social Security.

Supplementary Table 2: Average percentage distribution of the working-age population (15-69 years) by provinces of the Republic of Ecuador, 2016–2019

	%	(CI 95%)
**Ecuador**	**65.7**	**(65.0-66.4)**
Azuay	66.3	(65.5-67.1)
Bolívar	59.0	(58.3-59.6)
Cañar	62.2	(61.5-63.0)
Carchi	65.6	(64.8-66.3)
Cotopaxi	61.9	(61.1-62.6)
Chimborazo	63.5	(62.7-64.2)
El Oro	67.2	(66.5-68.0)
Esmeraldas	60.8	(59.9-61.7)
Galápagos	67.6	(67.5-67.7)
Guayas	67.5	(66.8-68.1)
Imbabura	64.8	(63.9-65.8)
Loja	63.6	(62.8-64.3)
Los Ríos	64.2	(63.4-64.9)
Manabí	65.3	(64.5-66.1)
Morona Santiago	57.8	(56.5-59.2)
Napo	60.2	(59.3-61.2)
Orellana	58.8	(58.1-59.6)
Pastaza	62.0	(60.9-63.1)
Pichincha	68.4	(67.9-68.9)
Santa Elena	63.2	(62.7-63.6)
Santo Domingo T.	65.0	(64.1-65.9)
Sucumbíos	63.6	(62.7-64.6)
Tungurahua	67.1	(66.4-67.7)
Zamora Chinchipe	59.8	(58.9-60.7)

Source: National Institute of Statistics and Census.
